# Auxin regulates anthocyanin biosynthesis through the Aux/IAA–ARF signaling pathway in apple

**DOI:** 10.1038/s41438-018-0068-4

**Published:** 2018-12-01

**Authors:** Yi-cheng Wang, Nan Wang, Hai-feng Xu, Sheng-hui Jiang, Hong-cheng Fang, Meng-yu Su, Zong-ying Zhang, Tian-liang Zhang, Xue-sen Chen

**Affiliations:** 1 State Key Laboratory of Crop Biology, College of Horticulture Science and Engineering, Shandong Agricultural University, Tai-An, Shandong China; 2 Collaborative Innovation Center of Fruit & Vegetable Quality and Efficient Production in Shandong, Tai-An, Shandong China

## Abstract

Auxin signaling, which is crucial for normal plant growth and development, mainly depends on ARF–Aux/IAA interactions. However, little is known regarding the regulatory effects of auxin signaling on anthocyanin metabolism in apple (*Malus domestica*). We investigated the functions of MdARF13, which contains a repression domain and is localized to the nucleus. This protein was observed to interact with the Aux/IAA repressor, MdIAA121, through its C-terminal dimerization domain. Protein degradation experiments proved that MdIAA121 is an unstable protein that is degraded by the 26S proteasome. Additionally, MdIAA121 stability is affected by the application of exogenous auxin. Furthermore, the overexpression of *MdIAA121* and *MdARF13* in transgenic red-fleshed apple calli weakened the inhibitory effect of MdARF13 on anthocyanin biosynthesis. These results indicate that the degradation of MdIAA121 induced by auxin treatment can release MdARF13, which acts as a negative regulator of the anthocyanin metabolic pathway. Additionally, yeast two-hybrid, bimolecular fluorescence complementation, and pull-down assays confirmed that MdMYB10 interacts with MdARF13. A subsequent electrophoretic mobility shift assay and yeast one-hybrid assay demonstrated that MdARF13 directly binds to the promoter of *MdDFR*, which is an anthocyanin pathway structural gene. Interestingly, chromatin immunoprecipitation–quantitative real-time PCR results indicated that the overexpression of *MdIAA121* clearly inhibits the recruitment of MdARF13 to the *MdDFR* promoter. Our findings further characterized the mechanism underlying the regulation of anthocyanin biosynthesis via Aux/IAA–ARF signaling.

## A green light for red apples

Insights into a hormonal signaling pathway controlling apple coloration could help growers to consistently produce eye-catching fruit. Auxins are signaling molecules that regulate many important metabolic pathways in plants. Research from a team led by Xue-sen Chen at Shandong Agricultural University in China now reveals how the auxin naphthalene acetic acid (NAA) controls production of anthocyanin, the molecule that gives red apples their distinctive coloration. The researchers determined that increasing NAA levels result in reduced production of anthocyanin. These effects are mediated by a subset of auxin-associated proteins that regulate the expression of genes that contribute to the synthesis of this pigment. The color of fruit can strongly influence consumer purchasing decisions, and this signaling pathway could help horticulturalists to ensure that apples retain their visual appeal even under inclement growing conditions.

## Introduction

Color is an important fruit quality index that influences the purchasing behavior of consumers to some degree^1^. As a type of secondary metabolite, anthocyanins constitute one of the main pigments responsible for fruit color^2,3^. Additionally, their positive effects on human health include enhancing vascular elasticity, preventing cardiovascular disease, and protecting the liver from damage^4–7^.

Three main protein families (MYB, bHLH, and WD40) regulate anthocyanin biosynthesis by forming the MYB–bHLH–WD40 complex^8,9^. The MYB transcription factors (TFs) belong to one of the largest plant TF families, with some members associated with anthocyanin biosynthesis having been isolated and identified in many plant species, including *Arabidopsis thaliana*, maize, strawberry, and grape^10–13^. In apple, the three genes encoding MYB activators (MYB10, MYB1, and MYBA) involved in anthocyanin biosynthesis are alleles^14^. Two MYB TF genes (*MdMYB1* and *MdMYBA*), which were first isolated from fruit skin, have been confirmed to be responsible for the accumulation of anthocyanin^15,16^. Furthermore, *MdMYB10* consists of a characteristic R6 rearrangement in its promoter that is associated with increased anthocyanin biosynthesis in red-fleshed apples^17^.

Anthocyanin biosynthesis is influenced by environmental stimuli (e.g., light and temperature) and plant hormones (e.g., auxin and jasmonate)^18–21^. Moreover, auxins (naphthalene acetic acid (NAA) or 2,4-dichlorophenoxyacetic acid (2,4-D)) regulate secondary metabolic pathways, including those related to phenylpropanoid, flavonoid, and anthocyanin metabolism^22–25^. An earlier study involving a carrot suspension culture revealed that 2,4-D strongly inhibits anthocyanin biosynthesis^20^. Additionally, the application of exogenous NAA and 2,4-D can decrease the anthocyanin contents of transgenic tobacco calli^24^. In apple, studies involving red-fleshed apple calli revealed that increasing auxin concentrations can significantly inhibit anthocyanin biosynthesis^26,27^. However, the role of the auxin signaling pathway during the regulation of anthocyanin biosynthesis is unclear.

Auxin (indole-3-acetic acid (IAA)) regulates plant development by inducing rapid cellular responses and changes in gene expression. Auxin response factors (ARFs) together with auxin/IAA (Aux/IAA) proteins are TFs that function as key regulators of auxin-responsive transcription in plants^28–30^. Current auxin signaling models indicate that auxin responses mainly depend on the interaction between the homologous C-terminal domains of Aux/IAA and ARF proteins^31,32^. When cellular auxin concentrations are low, ARF activators at the promoters of auxin-responsive genes are thought to be inactive because of their association with Aux/IAA repressors^29,31^. When auxin concentrations increase, the Aux/IAA repressors are recruited to auxin receptors and degraded via the ubiquitin–proteasome pathway^33,34^. This degradation enables ARF activators to repress/activate the expression of auxin-responsive genes^33^.

Most ARFs consist of an N-terminal DNA-binding domain (DBD), a middle region that functions as an activation domain (AD) or a repression domain (RD), and a C-terminal dimerization domain (CTD)^35^. ARFs can specifically bind to the TGTCTC auxin-response element (AuxRE) in the promoters of primary/early auxin-response genes^35^. In the last few years, several new details regarding the role of ARFs in plant growth and development have been revealed. For example, AtARF3 and AtARF4 are important for the development of reproductive and vegetative tissues^36,37^. Additionally, AtARF5 is involved in embryonic and vascular tissue development^38,39^, while OsARF8 mediates hypocotyl elongation and influences auxin homeostasis^40^. Meanwhile, SlARF7 may negatively regulate fruit set, whereas the upregulated expression of *SIARF10* suggests that the encoded protein positively affects fruit development^41,42^. Thus, ARFs exhibit dynamic functions in plants and have a central role in the hub of transcriptional networks.

In the present study, we investigated the effects of different auxin concentrations on anthocyanin biosynthesis in red-fleshed apple calli. We also characterized an *ARF* gene, *MdARF13*, and confirmed that the MdARF13-mediated auxin signaling pathway helps regulate anthocyanin biosynthesis in apple, thereby revealing a novel metabolic function for ARF proteins.

## Materials and methods

### Plant materials and hormone treatments

Calli induced as previously described were cultured on Murashige and Skoog (MS) medium supplemented with 2 μmol/L 6-benzylaminopurine and 5, 10, 20, or 40 μmol/L NAA^43^. The pH of all media was adjusted to 5.8 ± 0.1. The red-fleshed apple calli were incubated under a 16 h light/8 h dark photoperiod (light intensity: 1000–2000 lx) for 18 days before being harvested.

### Anthocyanin extraction and absorbance measurements

Harvested calli (0.5 g each) were ground to a powder in liquid nitrogen and then treated with 15 mL 1% (v/v) HCl-methanol for 24 h at 4 °C in darkness. The solutions were then centrifuged for 10 min at 8000 × *g*. The absorbance of the supernatants was measured at 530 nm using a UV-2450 spectrophotometer (Shimadzu, Kyoto, Japan).

### RNA isolation and quantitative real-time polymerase chain reaction

Total RNA was isolated using an RNAprep Pure Plant Kit (Tiangen, Beijing, China). First-strand complementary DNA (cDNA) was synthesized using the RevertAid First-Strand cDNA Synthesis Kit (Fermentas, St. Leon-Roth, Germany). Three replicates were prepared for each sample. Quantitative real-time polymerase chain reaction (qPCR) analyses were conducted using 50 ng/μL cDNA as the template, SYBR Green PCR Master Mix (TransGen Biotech, Beijing, China), and the iCycler iQ5 system (Bio-Rad, Hercules, CA, USA). Details regarding the qPCR primers are provided in Supplementary Table S1. *MdActin* was used as the internal control.

### Amino acid sequence analysis and phylogenetic tree construction

The ARF amino acid sequences were obtained from the NCBI (National Center for Biotechnology Information) database and aligned using the DNAMAN software (Lynnon Biosoft, USA). A phylogenetic tree was constructed according to the neighbor-joining method using the MEGA5.0 program.

### Red-fleshed apple callus transformation

The intact *MdARF13* coding sequence (CDS) was inserted into the pRI101 vector containing the 35S promoter and a *green fluorescent protein* (*GFP*) tag sequence to generate the 35S::*MdARF13*-*GFP* construct. Additionally, the intact *MdIAA121* CDS was inserted into the pCAMBIA1301 vector containing the 35S promoter and a *GFP* tag sequence to prepare the 35S::*MdIAA121*-*GFP* construct. The resulting recombinant vectors were transferred to *Agrobacterium tumefaciens* LBA4404 cells. Two-week-old calli grown in liquid medium were cocultured with *A. tumefaciens* LBA4404 cells carrying the 35S::*MdARF13*-*GFP* construct on MS medium containing 2 μmol/L NAA and 4 μmol/L 6-benzylaminopurine at 25 °C for 2 days in darkness. The calli were then transferred to fresh MS medium supplemented with 662 μmol/L carbenicillin and 74 μmol/L kanamycin to screen for transgene-carrying calli. To obtain calli cotransfected with *MdARF13+MdIAA121*, the same method was used, but calli were transferred to MS medium supplemented with 662 μmol/L carbenicillin, 74 μmol/L kanamycin, and 40 μmol/L hygromycin. *GFP*-overexpressing calli were used as controls.

### Yeast two-hybrid assay

Yeast (*Saccharomyces cerevisiae*) two-hybrid (Y2H) assays were conducted according to the manufacturer’s instructions (Clontech, Mountain View, CA, USA). The *MdARF13* CDS was inserted into the pGBKT7 vector (i.e., bait), while the *MdIAA7*, *MdIAA121*, *MdMYB9*, and *MdMYB10* CDSs were inserted into the pGADT7 vector (i.e., prey). All recombinant vectors were cotransformed into yeast strain Y2H Gold cells using the lithium acetate method. The cells were cultured on synthetic defined (SD) medium lacking leucine and tryptophan (SD/−Leu/−Trp). Putative transformants were transferred to SD medium lacking adenine, histidine, leucine, and tryptophane (SD/−Ade/−His/−Leu/−Trp; Clontech) with or without X-α-gal.

### Bimolecular fluorescence complementation assay

For a bimolecular fluorescence complementation (BiFC) assay, the *MdARF13* CDS was inserted into the pSPYNE-35S vector (with an *NYFP* tag sequence) to generate the *MdARF13*-*NYFP* recombinant vector. The *MdIAA121* and *MdMYB10* CDSs were inserted into the pSPYCE-35S vector (with a *CYFP* tag sequence) to generate the *MdIAA121*-*CYFP* and *MdMYB10*-*CYFP* recombinant vectors. *A. tumefaciens* LBA4404 cells were transformed with the recombinant vectors and then cultured in liquid medium until the optical density (600 nm) reached 0.6. Onion epidermal cells were treated with equal volumes of different combinations of *A. tumefaciens* strains. Onion cells were cultured at 23 °C for 48 h. The yellow fluorescence protein (YFP) signal was detected using a Zeiss CLSM-5 confocal laser scanning microscope (excitation wavelength of 488 nm).

### Pull-down assay

The *MdIAA121* and *MdMYB10* open reading frames were cloned into the pET32a vector containing a poly-histidine (HIS) tag sequence. The intact *MdARF13* CDS was ligated into the pGEX-4T-1 vector containing a glutathione *S*-transferase (GST) tag sequence. The recombinant vectors were inserted into *Escherichia coli* BL21 (DE3) cells (TransGen) to induce the production of fusion proteins. The resulting proteins were mixed together and then purified in columns using the HIS tag. The purified mixed proteins were subsequently analyzed by western blotting with anti-HIS or anti-GST antibodies (Abmart, Shanghai, China).

### Yeast one-hybrid analysis

Yeast one-hybrid (Y1H) assays were conducted using yeast strain Y187 cells (Clontech) according to the manufacturer’s instructions. *MdARF13* was cloned into the pGADT7 vector, while the *MdDFR* promoter was inserted into the pHIS2 vector. Different combinations of recombinant and empty vectors were cotransformed into yeast Y187 cells, and the interactions were examined on SD medium lacking Trp, Leu, and His (SD/−Trp/−Leu/−His) with an optimal concentration of 3-amino-1,2,4-triazole.

### Electrophoretic mobility shift assays

Electrophoretic mobility shift assay (EMSA) experiments were completed using an EMSA kit (Pierce, Rockford, IL, USA) and biotin-labeled probes. Briefly, biotin-labeled probes were incubated in 1× binding buffer (2.5% glycerol, 10 mM EDTA, 5 mM MgCl_2_, and 50 mM KCl) with or without proteins at 24 °C for 25 min. An unlabeled probe was added to the reactions.

### Chromatin immunoprecipitation–quantitative real-time PCR analysis

The chromatin immunoprecipitation (ChIP) experiment was completed using a modified version of a published method^44^. The ChIP Assay Kit (Upstate Biotechnology, Lake Placid, NY, USA) and anti-GFP antibody (Abmart) were used for cross-linking, the removal of cross-linkers, immunoprecipitation, and elution. The ChIP signal was quantified by qPCR as the percentage of the total input DNA. The experiment was repeated three times.

### Subcellular localization analysis

Protoplasts isolated from apple callus cells were prepared and transformed as described by Hu et al.^45^. Transformed apple protoplasts were stained with the DNA-specific dye 4′,6-diamidino-2-phenylindole, which highlights the location of the nucleus. The protoplasts from transgenic calli were observed by fluorescence microscopy.

### In vitro protein degradation assay

Protein degradation assays were used to detect the dynamic changes to MdIAA13 in vitro. The assay buffer contained 25 mM Tris-HCl, pH 7.5, 10 mM NaCl, 10 mM MgCl_2_, 5 mM dithiothreitol, 10 mM ATP, and 4 mM phenylmethylsulfonyl fluoride. Wild-type red-fleshed calli were treated with buffer, and the extract was incubated with the MdIAA13-His fusion protein for specific periods. The relative MdIAA121 abundance was detected by western blot using anti-His monoclonal antibodies.

## Results

### Anthocyanin content and related gene expression under different auxin concentrations

**Fig. 1:**
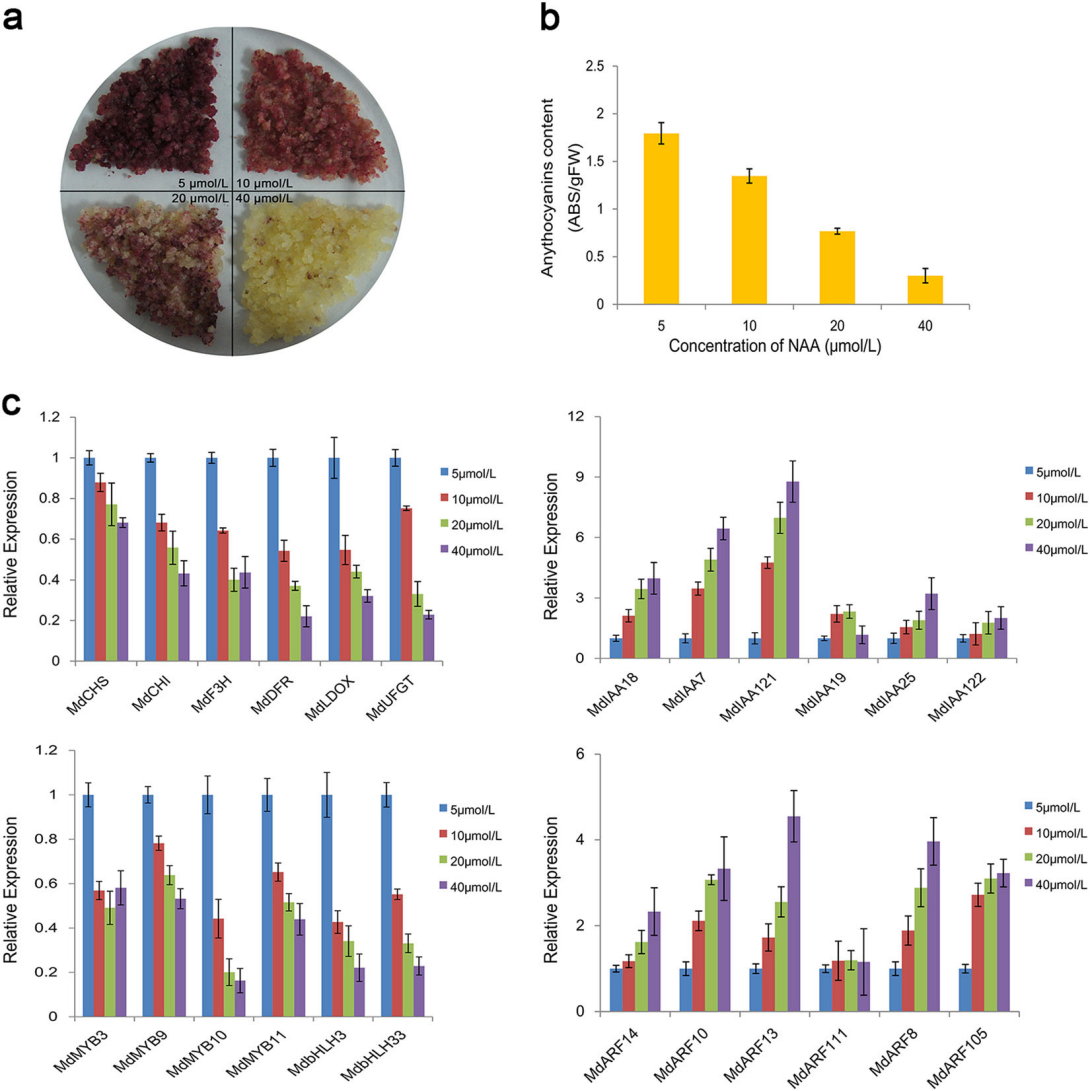
Red-fleshed apple calli on medium supplemented with different NAA concentrations (5, 10, 20, and 40 μmol/L). **a** Phenotypes of calli treated with different NAA concentrations, as well as the extraction of anthocyanin. **b** Relative anthocyanin content at 18 days. The relative anthocyanin content was calculated as follows: absorbance (530 nm)/fresh weight (g). **c** Relative expression levels of anthocyanin biosynthesis-related genes in response to different NAA treatments at 18 days

The anthocyanin contents of red-fleshed apple calli gradually decreased with increasing NAA concentrations (5–40 μmol/L), which caused the callus color to fade (Fig. 1a). Moreover, the anthocyanin content of calli cultured in 5  μmol/L NAA was 1.39-, 2.42-, and 5.98-fold higher than that of calli grown in 10, 20, and 40 μmol/L NAA, respectively (Fig. 1b). These results are consistent with the findings of Ji et al.^26,27^ and implied that low auxin concentrations were conducive to anthocyanin accumulation in red-fleshed apple calli.

The changes in the anthocyanin content of calli grown under different auxin conditions were also reflected at the transcript level. As the auxin concentration increased, the expression levels of anthocyanin biosynthesis-related genes decreased by varying degrees (Fig. 1c). However, the transcript levels for the *Aux/IAA* and *ARF* genes increased with increasing NAA concentrations (Fig. 1c). In particular, the *MdARF13* expression levels in calli treated with 40 μmol/L NAA were 2–5-fold higher than in calli exposed to the other NAA concentrations. This finding suggested that MdARF13 may repress anthocyanin biosynthesis.

### Bioinformatics analysis of the nuclear protein MdARF13

**Fig. 2:**
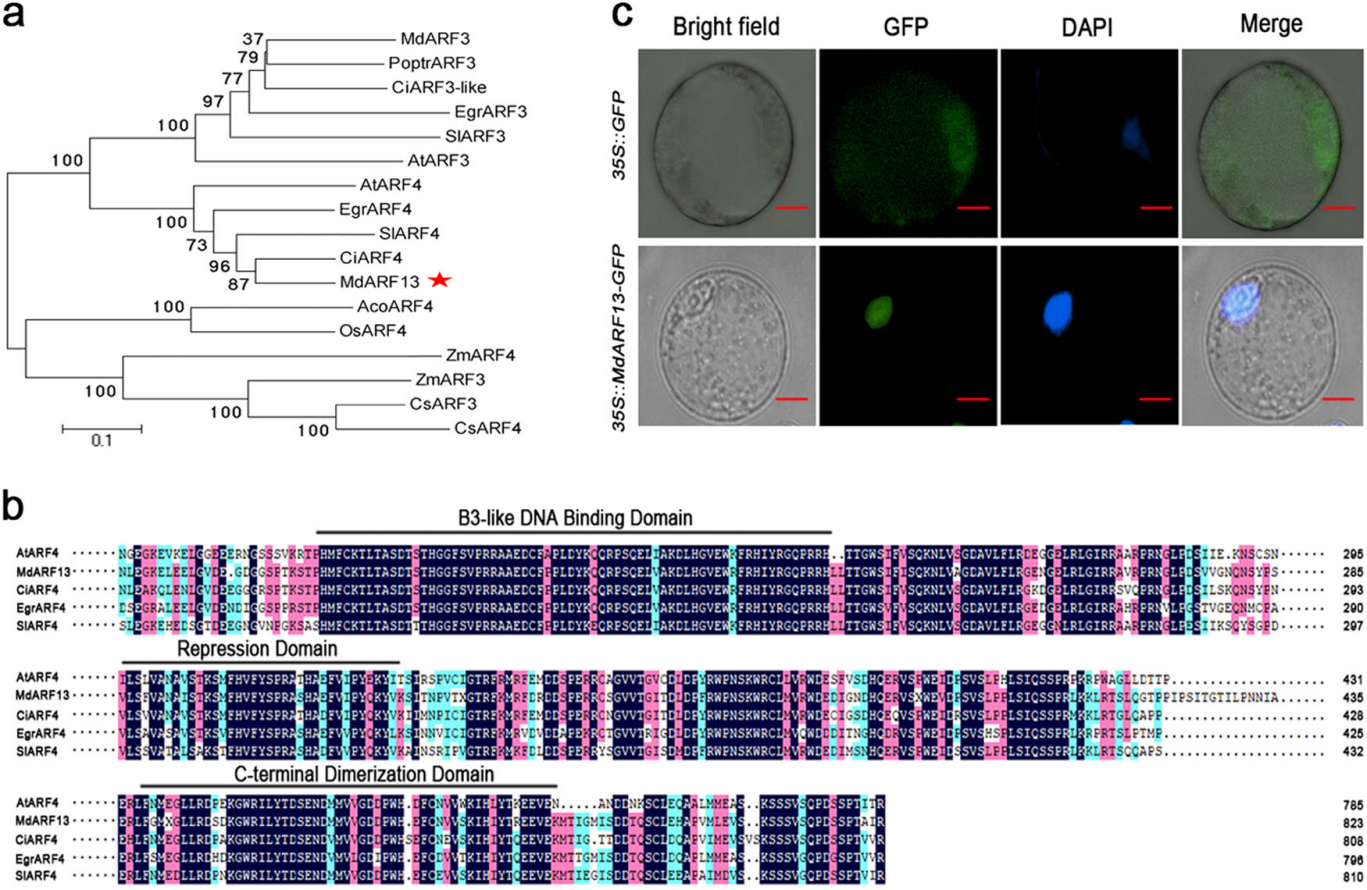
Bioinformatic analyses and MdARF13 subcellular localization. **a** Phylogenetic analysis of ARF transcription factors in different species. **b** Alignment of MdARF13, AtARF4, CiARF4, EgrARF4, and SiARF4 proteins. **c** Subcellular localization of the MdARF13-GFP fusion protein in transformed apple callus protoplasts. The protoplasts producing GFP alone were used as the control. Bars = 50 μm

Extensive bioinformatics and biochemical analyses of plants have clarified the number of *ARF* genes and their potential functions. In *A. thaliana*, experiments have revealed that AtARF4 contains an active RD and functions as a repressor in plant protoplast transfection assays^35^. A phylogenetic analysis to elucidate the relationship between MdARF13 and ARF proteins from other plant species indicated that MdARF13 and AtARF4 belong to the same clade (Fig. 2a). Additionally, the aligned protein sequences revealed that MdARF13 and ARF proteins from other plant species contain a conserved B3-like DBD, an RD, and a domain homologous to the Aux/IAA proteins (CTD) (Fig. 2b).

The subcellular localization of MdARF13 was studied by introducing a 35S::*MdARF13*-*GFP* construct into the protoplasts of red-fleshed apple calli. The transgenic calli carrying the empty vector (35S::GFP) were used as the control. In protoplasts expressing 35S::*MdARF13*-*GFP*, the GFP signal was observed only in the nucleus, while the GFP signal was detected throughout the control protoplasts (Fig. 2c), implying that MdARF13 is localized to the nucleus.

### MdARF13 interacts with MdIAA121

**Fig. 3:**
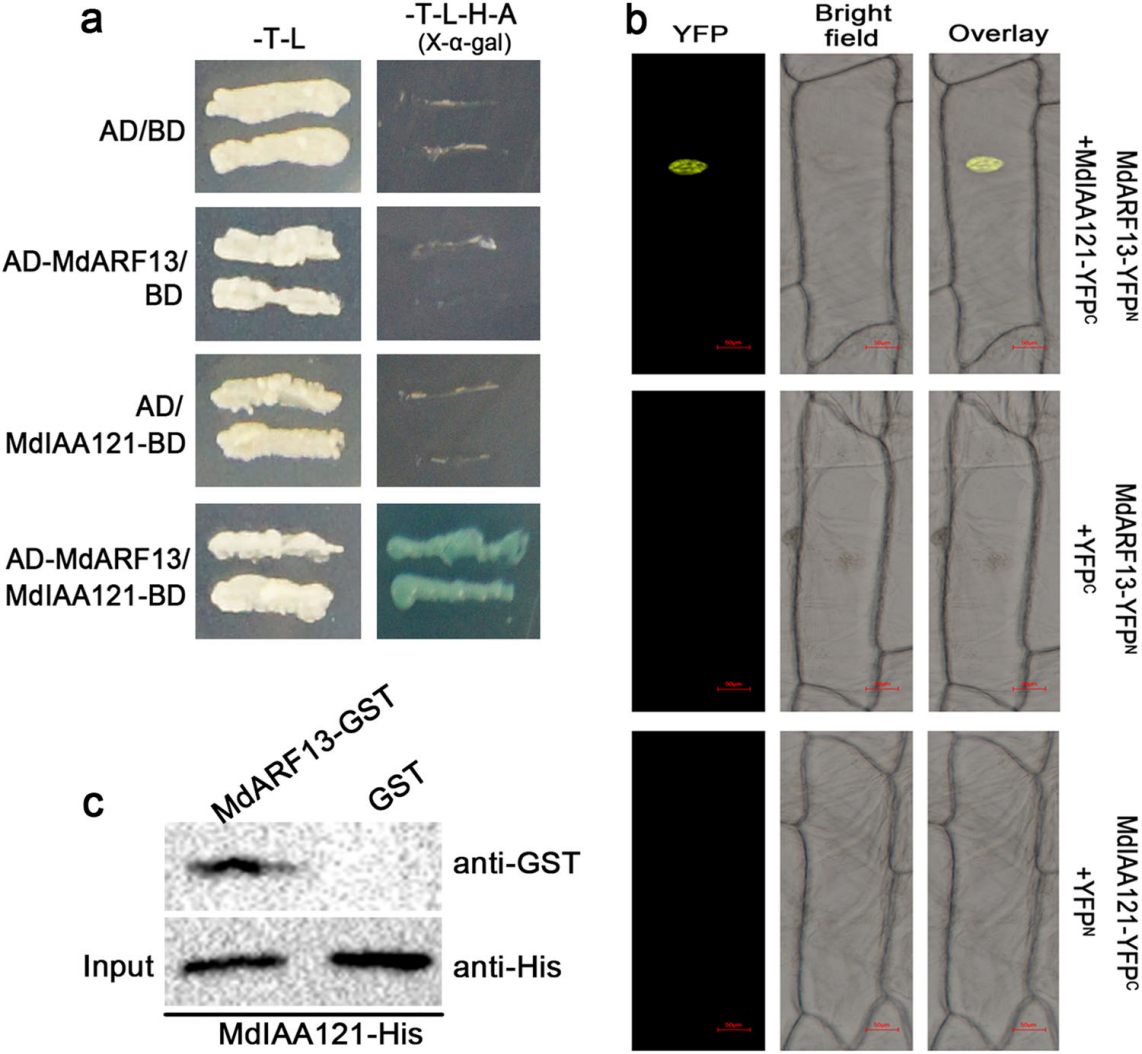
MdARF13 is targeted by MdIAA121. The interaction between MdARF13 and MdIAA121 was verified in **a** a yeast two-hybrid assay, **b** a bimolecular fluorescence complementation assay, and **c** a pull-down assay

The ARFs contain a dimerization domain responsible for the formation of heterodimers with Aux/IAA proteins^31^. The Y2H assays, which were used to test whether MdARF13 interacts with Aux/IAA proteins, revealed that MdARF13 interacts only with MdIAA121 (Fig. 3a). The BiFC assay involving onion epidermal cells cotransformed with *MdARF13-NYFP* and *MdIAA121-CYFP* constructs confirmed that MdARF13 interacts with MdIAA121 (Fig. 3b). The nuclei of these onion epidermal cells produced a strong YFP signal according to laser scanning confocal microscopy. In contrast, the YFP signal was not detected in cells in which MdIAA121-CYFP was replaced with CYFP alone. Meanwhile, our pull-down assays revealed that the recombinant MdIAA121-HIS fusion protein could be purified with MdARF13-GST, but not with GST alone (Fig. 3c). These results were consistent with those of the Y2H and BiFC assays. Our data suggested that MdARF13 can interact with MdIAA121 under in vivo and in vitro conditions.

### Auxin affects MdIAA121 stability

**Fig. 4:**
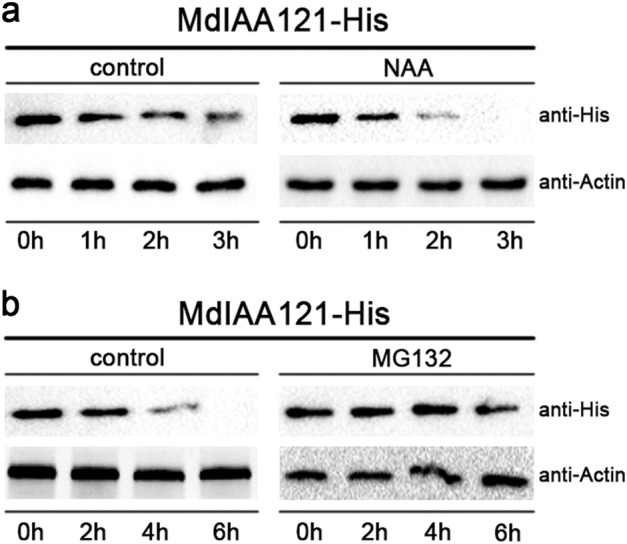
MdIAA13 is degraded by the 26S proteasome in the auxin signaling pathway. **a** A wild-type callus was treated with DMSO or 40 mmol NAA. Callus extracts were treated with the MdIAA121-His fusion protein and then incubated for the indicated periods. Actin was used as a loading control. **b** MG132 inhibited the degradation of MdIAA121. A wild-type apple callus extract was treated with DMSO or 100 mM MG132 for 0.5 h and then incubated with MdIAA121-His for the indicated periods

Ubiquitination, which represents an important posttranslational modification, is a key regulator of various processes, such as hormone signaling, responses to light and sugars, plant development, and plant immunity. In the Aux/IAA–ARF signaling pathway, auxin treatments promote the degradation of Aux/IAA by the 26S proteasome to release ARF proteins^46^. We conducted protein degradation assays to study the posttranslational regulation of MdIAA121 in vitro. The MdIAA121-His proteins were rapidly degraded after NAA was added (Fig. 4a). Furthermore, samples were treated with MG132 and dimethyl sulfoxide (DMSO). The MG132 treatment noticeably weakened the effect of auxin on MdIAA121 stability (Fig. 4b). These findings indicated that auxin induces the degradation of MdIAA121 via the 26S proteasome.

### Overexpression of *MdARF13* repressed anthocyanin accumulation in the apple callus

**Fig. 5:**
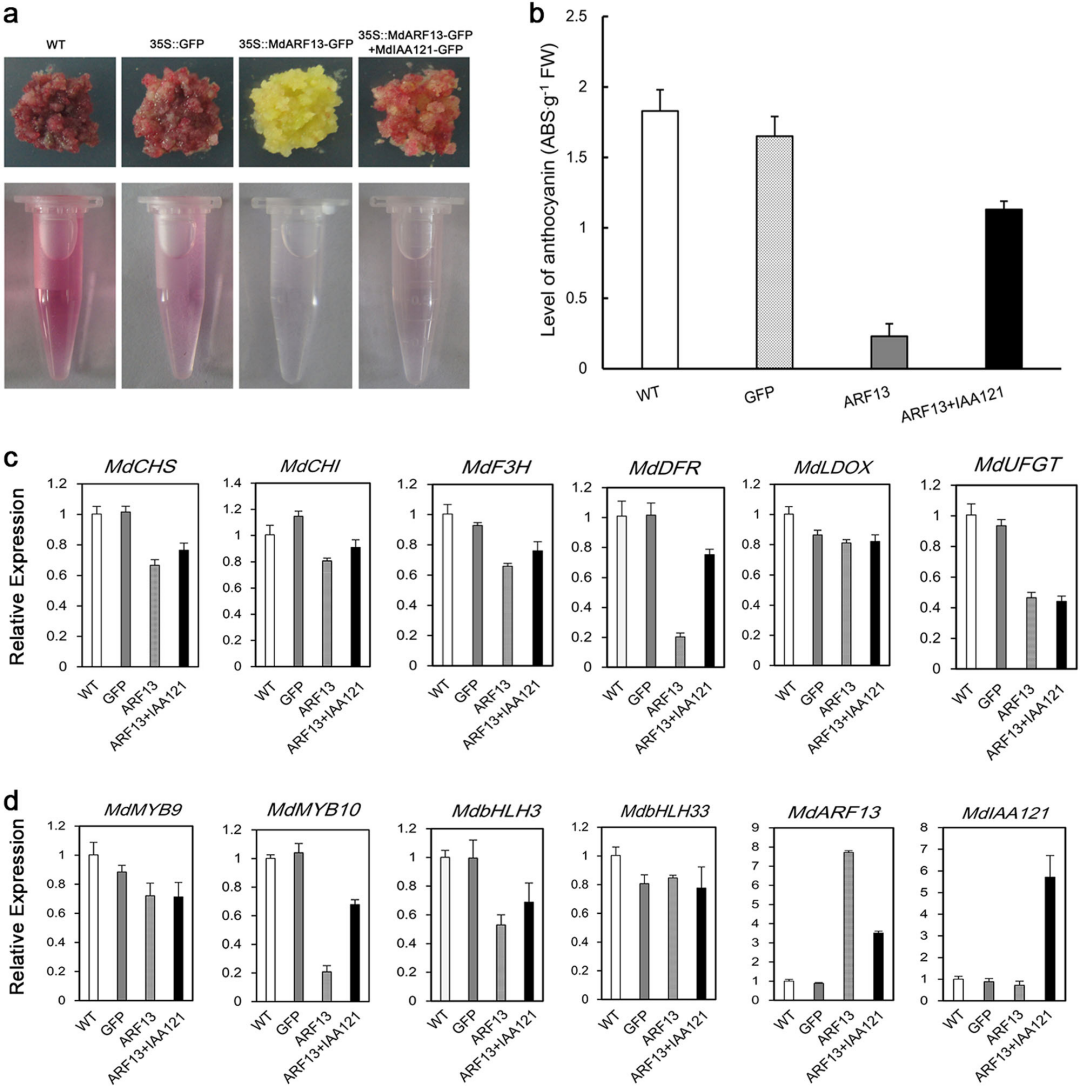
Analysis of transgenic red-fleshed apple calli. WT wild-type red-fleshed callus, 35S::GFP transgenic callus carrying the empty vector, 35S::*MdARF13*-*GFP* red-fleshed apple callus overexpressing *MdARF13-GFP,* 35S::*MdARF13*-*GFP*+*MdIAA121*-*GFP* red-fleshed apple callus overexpressing *MdARF13*-*GFP* and *MdIAA121*-*GFP*. **a** Appearance of the wild-type and transgenic red-fleshed apple calli. **b** Anthocyanin levels in wild-type and transgenic calli. **c**, **d** Expression of anthocyanin biosynthesis-related structural and transcription factor genes in wild-type and transgenic calli

To examine whether MdARF13 affects anthocyanin accumulation, *MdARF13* was overexpressed in red-fleshed apple calli. The color of the resulting transgenic calli changed from dark red to yellow, while the transcript levels of anthocyanin biosynthesis-related genes and the accumulation of anthocyanin decreased (Fig. 5). These observations implied that MdARF13 represses anthocyanin biosynthesis. To assess whether MdIAA121 inhibits MdARF13 activities, we generated calli cotransfected with *MdARF13+MdIAA121* by *A. tumefaciens*-mediated transformation. The expression levels of anthocyanin structural genes and anthocyanin content were obviously higher in cotransfected calli than in calli overexpressing *MdARF13* (Fig. 5). These results demonstrated that MdARF13 together with MdIAA121 mediate auxin signaling to regulate anthocyanin biosynthesis.

### MdARF13 interacts with MdMYB10

**Fig. 6:**
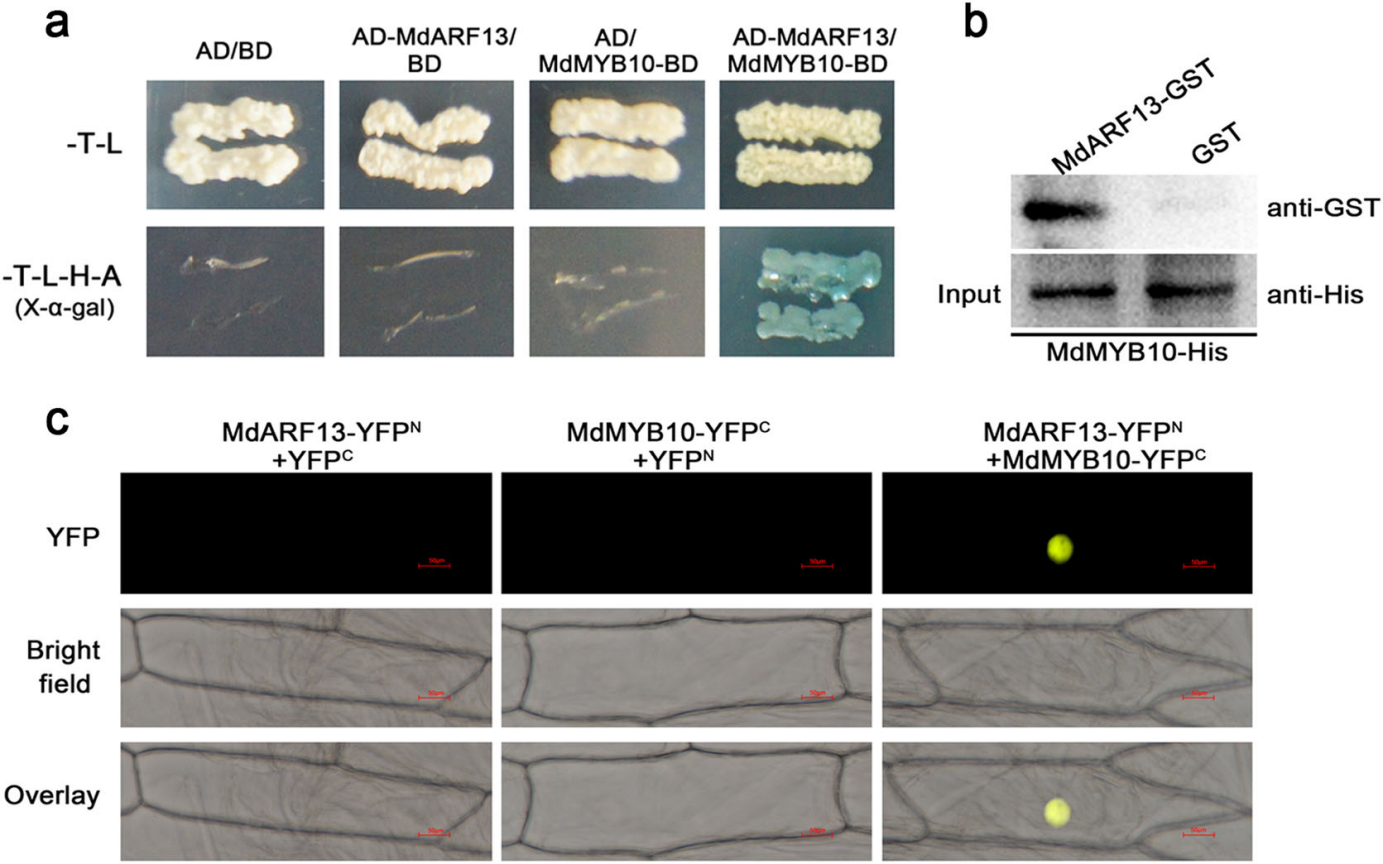
Interaction between MdARF13 and MdMYB10. The interaction between MdARF13 and MdMYB10 was verified in **a** yeast two-hybrid, **b** pull-down, and **c** bimolecular fluorescence complementation assays

The MYB TFs are crucial for anthocyanin biosynthesis, and the expression of the corresponding genes is regulated by the external environment. In this study, we hypothesized that MdARF13 is responsive to auxin and affects *MYB* expression. To verify this hypothesis, we completed Y2H assays to investigate whether MdARF13 interacts with MdMYB9 and MdMYB10. We observed that MdARF13 interacts only with MdMYB10 (Fig. 6a). The physical interaction between MdARF13 and MdMYB10 was further confirmed in pull-down and BiFC assays (Fig. 6b, c). Thus, we speculated that interactions between MdARF13 and MdMYB10 affect anthocyanin biosynthesis.

### MdARF13 binds to the *MdDFR* promoter

**Fig. 7:**
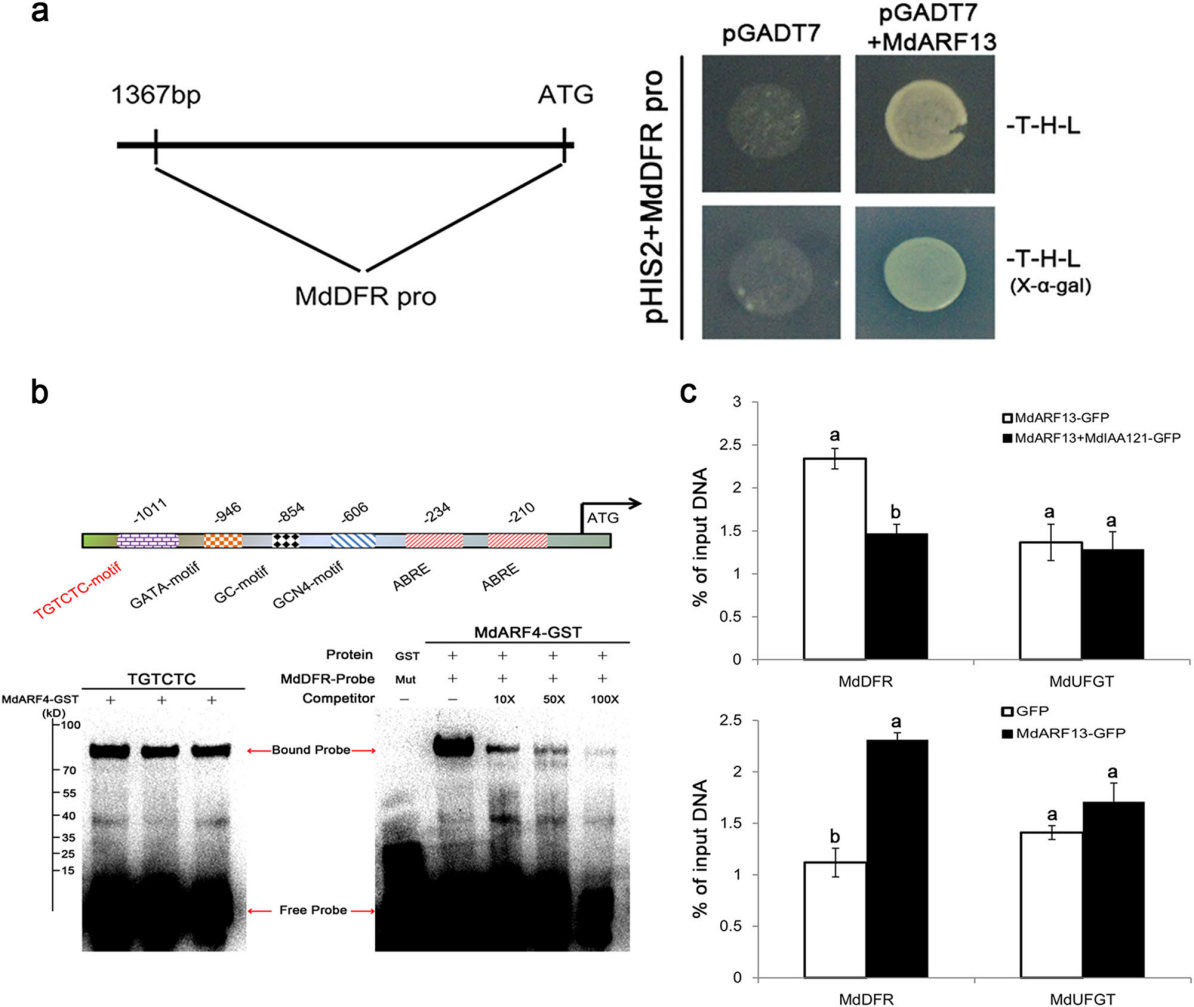
MdARF13 binds directly to the *MdDFR* promoter. **a** The 1427-bp *MdDFR* promoter sequence was analyzed. MdARF13 interacted with *MdDFR* promoter fragments in a Y1H assay. **b** Schematic diagram of the *MdDFR* promoter with an ARF-binding site (AuxRE). The EMSA results indicated that the MdARF13-GST fusion protein could bind directly to the AuxRE in the *MdDFR* promoter. Biotin-labeled probes were incubated with the MdARF13-GST fusion protein, and the free and bound DNA fragments were separated in an acrylamide gel. Unlabeled probes were used as competitors. **c** ChIP–qPCR data revealed that MdARF13 interacts with the *MdDFR* promoter in vivo, while MdIAA121 inhibits this interaction. The ChIP signal was quantified by qPCR as the percentage of the total input DNA. An actin gene was used as an internal control

ARF TFs regulate their downstream target genes by binding to the TGTCTC AuxRE in the promoter region and subsequently promoting or repressing expression^29^. We analyzed the promoter elements of the anthocyanin biosynthesis structural genes (*MdCHS*, *MdCHI*, *MdDFR*, *MdLDOX*, *MdFLS*, and *MdUFGT*) using the PlantCARE *cis*-acting regulatory element database (http://bioinformatics.psb.ugent.be/webtools/plantcare/html/) and determined that only *MdDFR* contains a putative AuxRE in its promoter region (Fig. 7b). We conducted an EMSA to clarify whether MdARF13 is recruited to the *MdDFR* promoter region. The assay results indicated that MdARF13 could interact with DNA probes containing the TGTCTC AuxRE (Fig. 7b). Moreover, Y1H assays confirmed that MdARF13 could interact with the *MdDFR* promoter (Fig. 7a), suggesting that MdARF13 suppresses the expression of *MdDFR* to decrease anthocyanin accumulation. Additionally, the ChIP–qPCR assay results suggested that the MdARF13-GFP fusion protein could bind to the *DFR* promoter, while the overexpression of *MdIAA121* in *MdARF13*-overexpressing calli clearly inhibited the recruitment of MdARF13 to the *MdDFR* promoter (Fig. 7c).

## Discussion

Auxin has long been recognized for its essential role in plant growth and development. Previous studies concluded that auxin signaling regulates anthocyanin biosynthesis in plant calli, which is associated with changes in the external auxin concentration^20,26^. In separate studies, Ji et al.^26^ and Liu et al.^47^ observed that increasing auxin concentrations within a certain range can inhibit anthocyanin biosynthesis in apple and *A. thaliana*. In this study, we observed that increasing NAA concentrations increased the inhibition of anthocyanin accumulation. Deikman and Hammer^48^ reported that the application of exogenous auxins suppressed the expression of anthocyanin regulatory and structural genes to varying degrees. Similarly, we observed that the expression levels of most structural and regulatory genes decreased in calli treated with NAA. Furthermore, the expression of genes encoding two types of TFs (Aux/IAA and ARF) associated with auxin signaling also increased to varying degrees, especially *MdARF13*. Thus, we speculate that these auxin signaling factors help regulate the expression of genes related to the anthocyanin biosynthesis pathway.

The Aux/IAA and ARF TFs are an integral part of the complex and elaborate auxin signaling pathway. Genome-wide analyses of ARF families have been completed in many plant species, including rice, tomato, and maize^49–51^. In *A. thaliana*, in addition to ARF3, 13, 17, and 23, the ARF family mainly comprises proteins with the following three parts: an N-terminal DBD, a middle region that functions as an AD or RD, and a CTD^29^. In this study, we determined that MdARF13 is homologous to AtARF4 and has a similar structure. ARFs exist in the nucleus for a relatively short period^29^. Consistent with this previous report, we observed that MdARF13 is a nuclear protein. Most Aux/IAAs function as repressors and are believed to dimerize with ARF activators via their CTDs. However, the Aux/IAA repressors are rapidly degraded as auxin concentrations increase, thereby impairing their inhibitory effect on early auxin-responsive genes^35^. Our Y2H, BiFC, and pull-down assays revealed that MdARF13 can interact with MdIAA121. Additionally, exogenous auxin treatment induces the degradation of MdIAA121 by the 26S proteasome, suggesting that MdIAA121 might serve as a labile repressor of MdARF13. However, future studies will need to identify the specific ubiquitination mechanisms associated with MdIAA121 degradation.

To further investigate the functions of MdARF13 during anthocyanin biosynthesis, *MdARF13* was overexpressed in red-fleshed apple calli. A previous study involving transfected protoplasts indicated that AtARF4 functions as a transcriptional repressor^35^. In the current study, qPCR data revealed that the transcript levels of auxin-regulated anthocyanin biosynthesis-related genes were down-regulated more in the calli with the 35S::*MdARF13*-*GFP* construct than in the wild-type control. Thus, MdARF13 is likely an inhibitor of anthocyanin biosynthesis in apple. Additionally, Aux/IAA can function as a repressor that dimerizes with the target ARF^52^. We observed that overexpressing *MdIAA121* in calli overexpressing *MdARF13* weakened the inhibitory effect of MdARF13 on anthocyanin biosynthesis, suggesting that MdARF13 may be targeted by MdIAA121. The interaction between these two proteins may attenuate the inhibitory effect of MdARF13 on anthocyanin biosynthesis.

The MYB TFs are crucial for regulating anthocyanin biosynthesis in apples. External environmental factors affect anthocyanin accumulation because the associated signaling elements regulate the expression of *MYB* genes. An earlier study indicated that the regulation of anthocyanin biosynthesis by MdMYB1 is responsive to light, with COP1 functioning as a negative regulator that degrades MdMYB1 in darkness^53^. The HY5 TF targets the *MYBD* promoter in cytokinin-induced signaling pathways, leading to the accumulation of anthocyanin^54^. In this study, Y2H, BiFC, and pull-down assays verified the in vitro and in vivo interaction between MdARF13 and MdMYB10, suggesting that MdARF13 might repress anthocyanin accumulation by down-regulating *MdMYB10* expression. Moreover, ARF TFs contain DNA-binding motifs and play key roles in the regulation of downstream genes through TGTCTC sequence-specific interactions with promoters^30^. We detected an MdARF13-binding site upstream of the *MdDFR* gene (Fig. 7a). EMSA, ChIP, and Y1H assays were conducted to confirm the specific binding of MdARF13 to the *MdDFR* promoter. We also proved that MdIAA121 functions as a repressor of the auxin signal and interacts with MdARF13 to inhibit the recruitment of MdARF13 to the *MdDFR* promoter. Therefore, in apple, exogenous auxins repress anthocyanin biosynthesis most likely through the MdIAA121–MdARF13 signal transduction pathway.

**Fig. 8:**
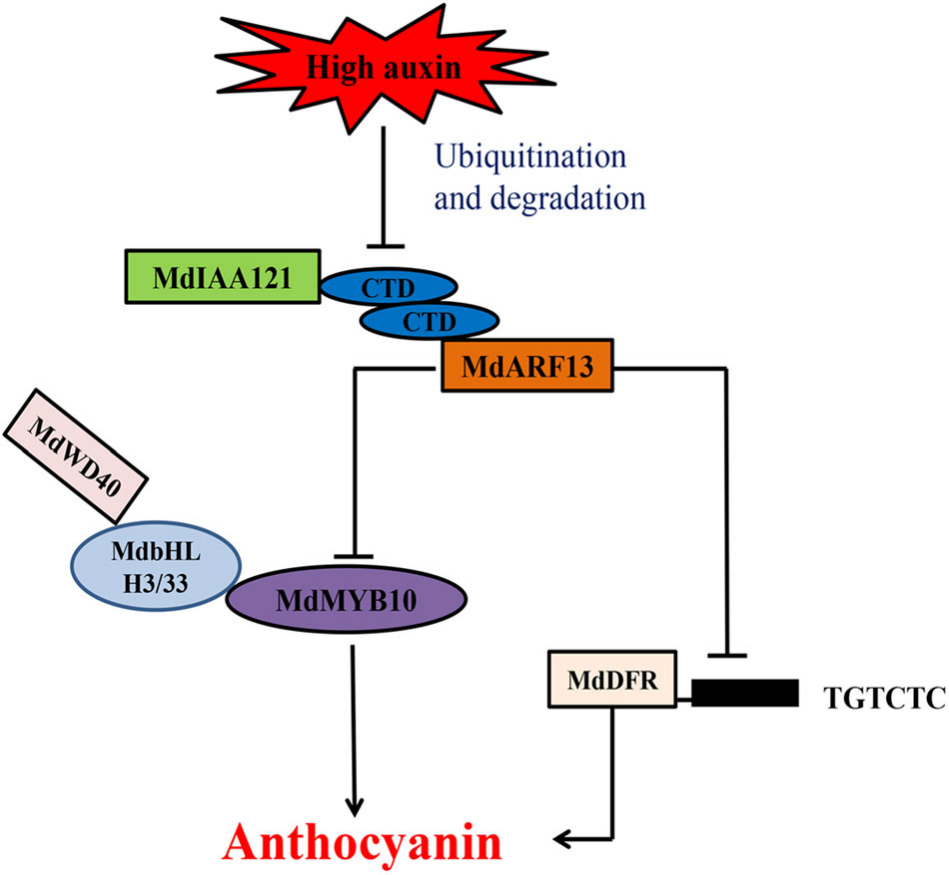
Model of the effects of auxins on anthocyanin accumulation via the Aux/IAA–ARF signaling pathway

ARF TFs are important for auxin signaling, and are involved in many processes related to plant growth and development. In this study, we observed that the inhibition of anthocyanin biosynthesis by high auxin concentrations involves MdARF13 (Fig. 8). Clarifying the role of auxin in the regulation of anthocyanin biosynthesis by ARFs may provide new insights into the regulation of anthocyanin metabolism by other hormones. During the cultivation of fruit trees, the rational application of hormones based on different environmental conditions can improve the appearance and nutritional value of fruit crops. Herein, we discussed the involvement of ARFs in the anthocyanin biosynthesis pathway, which may have implications for the development of new cultivation techniques aimed at improving fruit coloration under diverse environmental conditions.

## Electronic supplementary material

Table S1

Table S2
