# Supplementary material for: Auxin regulates anthocyanin biosynthesis through the Aux/IAA–ARF signaling pathway in apple
Source: Hortic Res. 2018 Dec 1;5:59. doi: 10.1038/s41438-018-0068-4 (PMC6269505; doi:10.1038/s41438-018-0068-4)
Supplement: Supplementary file 1 — Table S1 [file 41438_2018_68_MOESM1_ESM.docx]

| Gene name | Primer sequence of qRT-PCR |
| --- | --- |
| *MdActin* | F-5' TGACCGAATGAGCAAGGAAATTACT 3' |
|  | R-5' TACTCAGCTTTGGCAATCCACATC 3' |
| *MdCHS* | F-5' GGAGACAACTGGAGAAGGACTGGAA 3' |
|  | R-5' CGACATTGATACTGGTGTCTTC 3' |
| *MdCHI* | F-5' GGGATAACCTCGCGGCCAAA 3' |
|  | R-5' GCATCCATGCCGGAAGCTACAA 3' |
| *MdF3H* | F-5' TGGAAGCTTGTGAGGACTGGGGT 3' |
|  | R-5' CTCCTCCGATGGCAAATCAAAGA 3' |
| *MdDFR* | F-5' GATAGGGTTTGAGTTCAAGTA 3' |
|  | R-5' TCTCCTCAGCAGCCTCAGTTTTCT 3' |
| *MdLDOX* | F-5' CCAAGTGAAGCGGGTTGTGCT 3' |
|  | R-5' CAAAGCAGGCGGACAGGAGTAGC 3' |
| *MdUFGT* | F-5' CCACCGCCCTTCCAAACACTCT 3' |
|  | R-5' CACCCTTATGTTACGCGGCATGT 3' |
|  |  |

| Gene name | Primer sequence of qRT-PCR |
| --- | --- |
| *MdMYB3* | F-5' GAATCGGAGGCCAATTGCAG 3' |
|  | R-5' ATGATCCGCTGCAGTCTTGA 3' |
| *MdMYB9* | F-5' ACATTGATGCGAAGGGAG 3' |
|  | R-5' TAAATCCAACGAGGCACC 3' |
| *MdMYB10* | F-5' TGCCTGGACTCGAGAGGAAGACA 3' |
|  | R-5' CCTGTTTCCCAAAAGCCTGTGAA 3' |
| *MdMYB11* | F-5' GGCAACAGATGGTCTTTG 3' |
|  | R-5' TTCTTGGGTTGGTCTACG 3' |
| *MdbHLH3*  *MdbHLH33* | F-5' ACCACCTCAGCCAGAACCT 3'  R-5' CCTTCACCTTGGCTCTTAGTT 3'  F-5' ATGTTTTTGCAACGGAGAGAGCA 3'  R-5'TAGGCGAGTGAACACCATACATTAAAGG 3' |
|  |  |
| Gene name | Primer sequence of qRT-PCR |
| *MdARF14* | F-5' CAGCAACATACCAGAGAT 3' |
|  | R-5' CATATCACCTTCATCATCAG 3' |
| *MdARF10* | F-5' CTTCTCAGCAACATATCAG 3' |
|  | R-5' CATATCACCTTCATCATCAG 3' |
| *MdARF13*  *MdARF111*  *MdARF8*  *MdARF105* | F-5' GTTTTCTGTTCCTCGGAGA 3'  R-5' TTTCTTGATGATCATTTCCTATAT 3'  F-5' CAAGTACAGTTGCTCTAC 3'  R-5' CTCCTCCAGTTCATCTAT 3'  F-5'AGTCTCACCAGTTCTTAC 3'  R-5'CGTCTTCTGATTCCATAAAG 3'  F-5'GCCAAATAATGTCCTGATAC 3'  F-5' TGATGCCTCCAAGATTAC 3' |

| Gene name | Primer sequence of qRT-PCR |
| --- | --- |
| *MdIAA18* | F-5' AATAGTGGGGTGGCCACCAATC 3' |
|  | R-5' GACATGAACATATCCCATG 3' |
| *MdIAA7* | F-5' GATGGAAGGAGCAACTAG 3' |
|  | R-5' CATCTTTGCCTCACATTC 3' |
| *MdIAA121*  *MdIAA19*  *MdIAA25*  *MdIAA122*` | F-5' GAAGATGATACGGCAGTT 3'  R-5' CTCCACCAATAGTTGTAGT 3'  F-5' GAAGAACTTGATGAAGAGC 3'  R-5' GGTACATATTCCACTCCAT 3'  F-5' CCTCCAGTGTGTTCATAC 3'  R-5' TAGCATCCAGTCTCCATC 3'  F-5' CAATAACTGCCAGATGTC 3'  R-5' GATGTTCCAGATGATGAAG 3' |

| Gene name | Primer sequence of Y2H |
| --- | --- |
| *MdARF13* | F-5'CATATGATGGAAATTGATCTGAACCATGC3' |
|  | R-5’ GTCGACTTAGACCCGGATAGCCGTCGG 3' |
| *MdIAA121* | F-5' CATATGATGGAAGCCACACTTGGGTT 3' |
|  | R-5' GGATCCCTTCTTTCTGTTATCAAACTGTA3' |
| *MdMYB10* | F-5' CGGAATTCATGGAGGGATATAACG 3'  R-5' CGGGATCCTTCCTCTAACTCAATGC 3' |

| Gene name | Primer sequence of BIFC |
| --- | --- |
| *MdARF13* | F-5'ACTAGTATGGAAATTGATCTGAACCATGC3' |
| *MdIAA121*  *MdMYB10* | R-5' GTCGACGACCCGGATAGCCGTCGG 3'  F-5'ACTAGTATGGAAGCCACACTTGGGTTGC 3'  R-5' GTCGACCTTCTTTCTGTTATCAAACTGTA 3'  F-5' ATGGATCCATGGAGGGATATAACGAA 3' |
|  | R-5' TTGTCGACTTCTTCTTTTGAATGATTC 3' |
|  |  |

| Gene name | Primer sequence of pull-down |
| --- | --- |
| *MdARF13* | F-5' GCGTCGACATGGAAATTGATCTGAACC 3' |
|  | R-5’ CCTCGAGGACCCGGATAGCCGTC 3' |
| *MdIAA121* | F-5' GGATCCATGGGGAGAGCACCTTGT 3' |
|  | R-5'GAGCTCCTTCTTTCTGTTATCAAACTGTA 3' |
| *MdMYB10* | F-5'GGATCCATGGAGGGATATAACGAA 3'  R-5'GGATCCTTCTTCTTTTGAATGATTC 3' |

| Gene name | Primer sequence of Y1H |
| --- | --- |
| *MdDFR* | \| F-5'CGGAATTCGCTTTTGGTGAGAATTGAAC 3' \| \| --- \| \| R-5'CGAGCTCTTGAGTGTTTTTGTCAGCA 3' \| |
|  |  |
|  |  |

| Gene name | Primer sequence of transgenic callus |
| --- | --- |
| *MdARF13* | F-5' CCCATATGATGGAAATTGATCTGAACC3' |
|  | R-5’ GCGTCGACGACCCGGATAGCCGTC 3' |
| *MdIAA121* | F-5'CCCATATGATGGGGAGAGCACCTTGT 3'  R-5'GCGTCGACCTTCTTTCTGTTATCAAACTGTA 3' |
|  |  |

| Gene name | Primer sequence of EMSA |
| --- | --- |
| *MdDFR* | F-5' CGCCACATCATGTCTCTGACACAATA 3' |
|  | R-5’ TATTGTGTCAGAGACATGATGTGGCG 3' |
| *MdARF13*  Mutation | F-5' GCGTCGACATGGAAATTGATCTGAACC 3'  R-5' CCTCGAGGACCCGGATAGCCGTC 3'  F-5' CGCCACATCAAGTCTGTGACACAATA 3'  R-5’ TATTGTGTCACAGACTTGATGTGGCG 3' |
|  |  |
