# Supplementary material for: Auxin regulates anthocyanin biosynthesis through the Aux/IAA–ARF signaling pathway in apple
Source: Hortic Res. 2018 Dec 1;5:59. doi: 10.1038/s41438-018-0068-4 (PMC6269505; doi:10.1038/s41438-018-0068-4)
Supplement: Supplementary file 2 — Table S2 [file 41438_2018_68_MOESM2_ESM.docx]

The NCBI accession numbers of these transcription factors

| Transcription factors | NCBI accession numbers |
| --- | --- |
| PoptrARF3 | EEE85616.2 |
| CiARF3-like | XP_015382506 |
| EgrARF3 | XP_010052260.1 |
| SIARF3 | NP_001234316.1 |
| AtARF3 | AAB62404.1 |
| AtARF4  EgrARF4  SIARF4`  CiARF4  AcoARF4  OsARF4  ZmARF4  ZmARF3  CsARF3  CsARF4 | AAB62404.1  XP_010043718.1  NP_001233771.1  XP_006488135  XP_020090490.1  BAB85913.1  ADG43138.1  ADG43137.1  BAD19063.1  BAD19064.1 |
|  |  |
|  |  |

The GDR accession numbers of these transcription factors

| Transcription factors | GDR accession numbers |
| --- | --- |
| MdARF14 MDP0000929655  MdARF10 MDP0000190950  MdARF13 MDP0000412781 | |
| MdARF111 | MDP0000259062 |
| MdARF8  MdARF105  MdIAA121 | MDP0000310875  MDP0000876321  MDP0000131759 |
| MdIAA18 MDP0000303142  MdIAA7 MDP0000010086  MdIAA19 MDP0000124810  MdIAA25 MDP0000296324  MdIAA122 MDP0000267601 | |
